# Retrospective cohort study on glycemic control improvements after bariatric surgery among patients with type 2 diabetes

**DOI:** 10.6026/973206300213080

**Published:** 2025-09-30

**Authors:** Dhyaneshwar Ra, Poornima Venkatraman, Vishal Mruthyunjaya Kalmani, Yeruva Bheemeswara Reddy

**Affiliations:** 1 Department of General Surgery, Government Stanley Medical College Chennai, Tamil Nadu, India; 2 Department of Surgery, Davao Medical School Foundation, Philippines; 3 Department of Surgery, Barking Havering and Redbridge University Hospitals NHS Trust, Essex, United Kingdom; 4 Department of Surgery, Santhiram Medical College, Andhra Pradesh, India

**Keywords:** Bariatric surgery, type 2 diabetes, glycemic control, HbA1c, metabolic improvement, retrospective cohort

## Abstract

The impact of bariatric surgery on glycemic control in 128 patients with type 2 diabetes over a 12-month postoperative period is of interest. Pre- and post-surgery glycemic markers, including HbA1c and fasting plasma glucose, were analyzed. Significant reductions in HbA1c levels were observed, with 68% of patients achieving remission or requiring reduced medication. Improvements were more pronounced in patients with shorter diabetes duration and greater weight loss. Thus, we show bariatric surgery as an effective intervention for metabolic control in type 2 diabetes.

## Background:

Type 2 diabetes mellitus (T2DM) is a chronic metabolic disorder characterized by insulin resistance and progressive pancreatic β-cell dysfunction [1]. It is strongly associated with obesity, which exacerbates insulin resistance and complicates glycemic control [2]. Traditional management strategies-including lifestyle modification, oral hypoglycemics, and insulin therapy-often fail to achieve sustained glycemic targets, particularly in patients with severe obesity [3]. Bariatric surgery has emerged as a powerful metabolic intervention beyond weight loss [4]. Several studies have demonstrated that procedures such as Roux-en-Y gastric bypass (RYGB) and sleeve gastrectomy lead to rapid and substantial improvements in glucose metabolism, even before significant weight loss occurs [5]. These effects are mediated through mechanisms like enhanced incretin response, gut hormone modulation, and improved insulin sensitivity [6]. Glycemic remission or substantial improvement post-bariatric surgery has been consistently documented in prospective trials, but data from real-world clinical settings-particularly long-term, retrospective cohorts-are still needed [7]. With an emphasis on HbA1c reductions, remission rates, and medication reliance over a 12-month period, this retrospective cohort study attempts to evaluate the effect of bariatric surgery on glycemic control in individuals with type 2 diabetes. Predictive factors linked to improved postoperative glycemic outcomes are also investigated in this study. Therefore, it is of interest report on glycemic control improvements after bariatric surgery among patients with type 2 diabetes.

## Materials and Methods:

In a tertiary care facility, this retrospective cohort study examined the medical records of individuals with type 2 diabetes mellitus (T2DM) who had bariatric surgery between January 2021 and December 2022. According to the availability of comprehensive preoperative and 12-month postoperative hyperglycemia data, 128 patients in total were included. Adults with T2DM diagnosed for at least a year prior to surgery, ages 18 to 65, met the inclusion criteria. Incomplete follow-up, prior bariatric surgeries and type 1 diabetes were among the exclusion criteria. Clinical indicators (BMI, length of diabetes, comorbidities), demographic factors (age, sex), and surgical information (kind of procedure: sleeve gastrectomy or Roux-en-Y gastric bypass) were among the data that were taken from electronic health records. Preoperative and postoperative measurements of HbA1c, fasting plasma glucose (FPG), body weight, and antidiabetic medication use were recorded. Primary outcomes were the change in HbA1c and FPG at 12 months postoperatively. Secondary outcomes included the rate of partial or complete diabetes remission, defined as HbA1c <6.5% without pharmacologic therapy for at least 6 months. Medication burden was categorized as none, oral agents only, or insulin-dependent. Descriptive statistics were used to summarize baseline characteristics. Paired t-tests were performed to compare pre- and post-surgery glycemic markers. Chi-square tests assessed changes in medication usage. Multivariate linear regression identified predictors of greater HbA1c reduction. A p-value of <0.05 was considered statistically significant. All analyses were performed using SPSS version 27.

## Results:

A total of 128 patients (58.6% female) with type 2 diabetes underwent bariatric surgery, with 79 receiving sleeve gastrectomy and 49 undergoing Roux-en-Y gastric bypass. Significant improvements in glycemic control were observed 12 months postoperatively. Mean HbA1c dropped from 8.4% to 6.3%, and 68.7% of patients achieved complete or partial diabetes remission. Patients with a shorter duration of diabetes and greater BMI reduction demonstrated more favorable outcomes. Table 1 (see PDF) shows the baseline characteristics show a moderately obese cohort with a high mean HbA1c and a wide range in diabetes duration. Table 2 (see PDF) shows majority of patients underwent sleeve gastrectomy, with fewer undergoing Roux-en-Y gastric bypass. Table 3 (see PDF) shows postoperative weight loss was substantial in both groups, with greater loss observed in RYGB recipients. Table 4 (see PDF) shows HbA1c levels significantly declined postoperatively in both surgical groups, more so in RYGB. Table 5 (see PDF) shows fasting plasma glucose also showed a significant reduction postoperatively. Table 6 (see PDF) shows nearly 69% of patients achieved partial or complete remission of diabetes by 12 months. Table 7 (see PDF) shows medication dependency significantly decreased, with many patients discontinuing insulin and/or oral agents. Table 8 (see PDF) shows shorter diabetes duration was associated with a higher rate of remission. Table 9 (see PDF) shows patients with greater BMI reduction also had greater HbA1c reduction. Table 10 (see PDF) shows multivariate regression identified BMI reduction, shorter diabetes duration, and pre-op HbA1c as independent predictors of greater glycemic improvement.

## Discussion:

This retrospective cohort study reinforces the growing body of evidence that bariatric surgery significantly improves glycemic control in patients with type 2 diabetes mellitus (T2DM). A 12-month follow-up revealed that bariatric surgery, particularly Roux-en-Y gastric bypass (RYGB), leads to marked reductions in HbA1c and fasting plasma glucose, with nearly 69% of patients achieving partial or complete remission of diabetes. These findings support the metabolic impact of bariatric procedures, which appear to go beyond weight loss alone [8]. The higher remission rates in patients undergoing RYGB compared to sleeve gastrectomy may be attributed to greater hormonal alterations, including enhanced GLP-1 secretion and improved insulin sensitivity. Furthermore, patients with a shorter duration of diabetes (<5 years) were more likely to achieve remission, underscoring the importance of early surgical intervention before irreversible pancreatic β-cell decline occurs [9]. The observed relationship between the degree of BMI reduction and HbA1c improvement highlights the additive value of weight loss in glycemic management, although hormonal mechanisms likely play a parallel role. Another important observation was the significant reduction in medication burden, particularly insulin discontinuation [10]. Participants had to be between the ages of 18 and 65 and have been diagnosed with type 2 diabetes for at least a year before surgery. Among the exclusion criteria were insufficient follow-up, prior bariatric surgeries and type 1 diabetes [11]. Data were taken from electronic health records and included clinical characteristics (BMI, length of diabetes, comorbidities), demographic variables (age, sex) and surgical information (kind of procedure: Roux-en-Y gastric bypass or sleeve gastrectomy). Because it is retrospective, it depends on the completeness and accuracy of collected data and is prone to selection bias [12]. Furthermore, the follow-up period was only 12 months; longer-term data are required to evaluate the sustainability of remission and glycemic changes. Although they were not recorded, variables including psychological state, physical activity and dietary compliance may have an impact on the results of surgery [13]. The study provides useful empirical data on the glycemic advantages of bariatric surgery in people with type 2 diabetes in spite of these drawbacks. All things considered, bariatric surgery is a very successful method of enhancing glycemic control in type 2 diabetes, especially when done early in the course of the disease [14]. Outcomes are mostly determined by the surgical method and the patient, particularly the length of diabetes and weight loss. For carefully chosen patients with obesity and type 2 diabetes, these findings lend credence to the inclusion of bariatric surgery in the treatment plan.

## Conclusion:

In patients with type 2 diabetes, bariatric surgery dramatically improves glycemic control; around 69% of patients experience remission or a decrease in the requirement for medication. Greater BMI reduction and shorter duration of diabetes were both highly predictive of improved outcomes. For suitable individuals with type 2 diabetes and obesity, these results corroborate early surgical surgery as an efficient metabolic therapy.
